# Human habitat modification, not apex scavenger decline, drives isotopic niche variation in a carnivore community

**DOI:** 10.1007/s00442-024-05544-9

**Published:** 2024-04-15

**Authors:** Olivia Bell, Menna E. Jones, Manuel Ruiz-Aravena, David G. Hamilton, Sebastien Comte, Rowena Hamer, Rodrigo K. Hamede, Jason Newton, Stuart Bearhop, Robbie A. McDonald

**Affiliations:** 1 Environment and Sustainability Institute, University of Exeter, Penryn, TR10 9FE UK; 2 School of Natural Sciences, University of Tasmania, Hobart, TAS 7005 Australia; 3 Department of Public and Ecosystem Health, Cornell University, Ithaca, NY 14850 USA; 4 Tasmanian Land Conservancy, 183 Macquarie Street, Hobart, TAS 7007 Australia; 5 Vertebrate Pest Research Unit, NSW Department of Primary Industries, 1447 Forest Road, Orange, NSW 2800 Australia; 6 National Environmental Isotope Facility, Scottish Universities Environmental Research Centre, East Kilbride, G75 0QF UK; 7 Centre for Ecology and Conservation, University of Exeter, Penryn, TR10 9FE UK

**Keywords:** Human-modified habitat, *Sarcophilus harrisii*, Stable isotope, Tasmanian devil, Trophic cascade

## Abstract

Top carnivores can influence the structure of ecological communities, primarily through competition and predation; however, communities are also influenced by bottom-up forces such as anthropogenic habitat disturbance. Top carnivore declines will likely alter competitive dynamics within and amongst sympatric carnivore species. Increasing intraspecific competition is generally predicted to drive niche expansion and/or individual specialisation, while interspecific competition tends to constrain niches. Using stable isotope analysis of whiskers, we studied the effects of Tasmanian devil *Sarcophilus harrisii* declines upon the population- and individual-level isotopic niches of Tasmanian devils and sympatric spotted-tailed quolls *Dasyurus maculatus* subsp. *maculatus.* We investigated whether time since the onset of devil decline (a proxy for severity of decline) and landscape characteristics affected the isotopic niche breadth and overlap of devil and quoll populations. We quantified individual isotopic niche breadth for a subset of Tasmanian devils and spotted-tailed quolls and assessed whether between-site population niche variation was driven by individual-level specialisation. Tasmanian devils and spotted-tailed quolls demonstrated smaller population-level isotopic niche breadths with increasing human-modified habitat, while time since the onset of devil decline had no effect on population-level niche breadth or interspecific niche overlap. Individual isotopic niche breadths of Tasmanian devils and spotted-tailed quolls were narrower in human-modified landscapes, likely driving population isotopic niche contraction, however, the degree of individuals’ specialisation relative to one another remained constant. Our results suggest that across varied landscapes, mammalian carnivore niches can be more sensitive to the bottom-up forces of anthropogenic habitat disturbance than to the top-down effects of top carnivore decline.

## Introduction

Top carnivores play a significant role in structuring ecological communities and ecosystems, but many species have undergone widespread decline and local extirpations (Estes et al. 2011; Ripple et al. 2014). By exerting predation pressure on prey, and competitive pressure upon mesopredators, top carnivores create a ‘landscape of fear’, influencing the abundance and behaviours of sympatric species (Schmitz et al. 1997; Ritchie and Johnson 2009; Laundré et al. 2010). Carnivore declines and losses can therefore cause trophic cascades with ecosystem-scale consequences (Elmhagen and Rushton 2007; Elmhagen et al. 2010). The nature and extent of ecosystem effects of top carnivores can be highly context-dependent and are influenced by a range of factors, including anthropogenic landscape modification (Kuijper et al. 2016), habitat fragmentation (Wang et al. 2020), landscape productivity (Elmhagen and Rushton 2007) and food web complexity (Finke and Denno 2004).

Shifts in the abundance and behaviours of sympatric species following carnivore declines will alter the strength of competitive interactions within communities. As competition is a key driving force in the realisation of ecological niches, including the diversity of resources exploited by populations (Roughgarden 1972), shifts in community structure can be expected to have consequences for niche partitioning at the level of individuals, populations and communities. Theory and empirical evidence suggest that increasing intraspecific competition drives niche expansion at the population level, generally via increasing variation among individuals as a result of individual specialisation, while interspecific competition constrains population niches (Van Valen 1965; Roughgarden 1972; Bolnick et al. 2003, 2010; Svanbäck and Bolnick 2007). Top carnivore decline could therefore result in shifting niche dynamics among competing species, potentially driving trophic cascades, for example, if mesopredator or prey populations are more productive following ecological release. However, trophic niches can also be shaped by bottom-up forces, including habitat and ecosystem fragmentation (Estes et al. 2003; Layman et al. 2007; Tinker et al. 2008; Newsome et al. 2009; Newsome et al. 2015a, b). For example, in the Great Lakes region of North America, increased availability of anthropogenic food resources in increasingly disturbed landscapes has driven increases in trophic niche breadth and niche overlap within a guild of seven carnivore species (Manlick and Pauli 2020).

We examine the effects of carnivore decline and landscape context upon the trophic niches of sympatric marsupial carnivores in Tasmania, Australia: Tasmanian devils *Sarcophilus harrisii*, spotted-tailed quolls *Dasyurus maculatus* subsp*. maculatus* and, where scarcer data permit, eastern quolls *Dasyurus viverrinus*. Tasmanian devils are the top terrestrial predator and scavenger in Tasmanian ecosystems (Cunningham et al. 2018; Jones, 2003). Devil populations have declined markedly as a result of Devil Facial Tumour Disease (DFTD), a largely fatal transmissible cancer that was first recognised in 1996 (Hawkins et al. 2006). Emerging in north-east Tasmania, DFTD has since spread south and west to cover approximately 80% of the devil’s modern range, causing populations to decline by an average of 80% in affected areas (Lazenby et al. 2018; Cunningham et al. 2021). The DFTD epizootic has resulted in a spatial gradient of devil abundance, which we use to study the responses of Tasmanian carnivores to declines in top carnivore populations.

Tasmanian devil declines have had cascading ecological effects. Where devils have declined, feral cat *Felis catus* abundance has increased (Hollings et al. 2014). Temporal partitioning of carnivore activity has changed; where devils are now living at low densities, devil activity has shifted to later in the evening, presumably due to decreased intraspecific competition reducing the need for individuals to search for resources early in high-density populations (Cunningham et al. 2019). In response, spotted-tailed quoll activity has shifted forward to early evening, suggesting competitive release from temporal avoidance behaviours. Where devils have declined, spotted-tailed quolls have increased their scavenging behaviour, discovering carcasses sooner and consuming them for longer (Cunningham et al. 2018), and consuming more large mammals, including common wombats *Vombatus ursinus* and Bennett’s wallaby *Macropus rufogriseus*, almost certainly via scavenging (Andersen et al. 2017). Little is known, however, about the impact of devil decline on the devil or spotted-tailed quoll trophic niches at the population level, and nothing is known about individual variation in trophic behaviour in response to devil decline. Increased scavenging behaviour in spotted-tailed quolls could reflect niche expansion or a niche shift with decreasing interspecific competition from devils. Based on the niche variation hypothesis (Van Valen 1965), we predict that areas where devils have been in decline the longest will have seen niche expansion in spotted-tailed quolls. For Tasmanian devils, the niche variation hypothesis would predict population-level niches are either similar or relatively larger in the remaining high-density populations, possibly with increased levels of individual specialisation (Van Valen 1965). Having said this, the relative effects of devil decline and landscape context on Tasmanian carnivores have not been explicitly compared, and bottom-up influences such as habitat composition and structure may disrupt the relationships predicted above. Recent evidence from a restricted area of the high rainfall north-west Tasmanian region suggests Tasmanian devil group isotopic niches are narrower in areas with human disturbance (Lewis et al. 2023), but this effect has not yet been demonstrated in other sympatric carnivores or across the breadth of the devil’s Tasmanian range, where the histories of disease and population decline vary markedly. The relationship between individual niche variation and human-disturbed landscapes has not been explored. It remains unclear what effect human-modified landscapes may have on the trophic ecology of sympatric carnivores, in this case Tasmanian marsupials, relative to potential top-down impacts of top predator decline.

Conventional dietary analyses are excellent at identifying the ranges of prey items consumed but are often snapshots, and less suitable for quantifying variation within and among individual animals’ diets. Stable isotope analysis allows the characterisation of aspects of the trophic ecology of both populations and individuals (Bearhop et al. 2004; Newsome et al. 2007). Ratios of heavy to light isotopes in consumer tissues broadly reflect those of their food sources, with predictable changes associated with the processing and incorporation of dietary proteins (DeNiro and Epstein 1978; Hobson and Clark 1992; Bearhop et al. 2002). Sampling consumer tissues for stable isotope analysis can be used to generate information on a wide range of individuals, and also to build a time series of data for individuals through repeat sampling (either via repeated sampling of rapidly integrating tissues such as blood, or more often, by subsampling continually growing tissues that become inert after formation, such as whiskers). Ratios of ^15^N to ^14^N (expressed as δ^15^N) within consumer tissues broadly reflect the trophic level of organisms, as the change in δ^15^N between food source tissue and consumer tissues is predictable (DeNiro and Epstein 1981). Variation in carbon ratios (^13^C to ^12^C, expressed as δ^13^C), on the other hand, tends to reflect the biochemical pathways of producers, largely the photosynthetic pathways of plants, and can provide information on foraging locations and habitats, as well as food sources consumed (Gannes et al. 1998). Isotopic niches are not equivalent to trophic niches, and should not be interpreted as such, however, with considered application, they can provide a robust means of quantifying variation. For example, stable isotope analysis has been applied to demonstrate variation in individual foraging specialisation within European badger *Meles meles* groups (Robertson et al. 2014), and increased individual specialisation with increasing group size in group-living banded mongooses *Mungos mungo* (Sheppard et al. 2018)*.* In Tasmanian devil research, stable isotope analysis has previously been used to demonstrate isotopic niche variation relating to age (Bell et al. 2020) and body size (Lewis et al. 2022), and to explore the trophic impacts of DFTD infection (Bell et al. 2021).

We apply stable isotope analysis to characterise the trophic ecology of devils and quolls at 5 study sites across Tasmania. We investigate the impact of devil decline and other ecological variables, including human-modified habitat coverage, on devil and quoll niches at the level of populations and individuals. To investigate isotopic niches at the population-level, we characterised the local sampled population isotopic niche breadths of Tasmanian devils and spotted-tailed quolls at each site, alongside the local sampled population isotopic niche breadths of eastern quolls at one site, and quantified the extent of overlap between species at each site. We then analysed the potential drivers of variation in isotopic niche breadth and overlap of the two main species, including the time since DFTD arrival (as a proxy for the severity of devil decline), the amount of human-modified habitat in the landscape, prey community assemblages and other extrinsic variables. To investigate niche change at the individual-level, we quantified the individual isotopic niche breadths of a subset of individual devils and spotted-tailed quolls. We then quantified their relative isotopic niche breadth as a proportion of their local sampled population isotopic niche breadth. Henceforth we refer to locally sampled population isotopic niche breadth as the population isotopic niche, although we have not sampled the entire local population. We analysed whether variation in population isotopic niche breadth among sites is driven by variation in individual specialisation, or individual generalism.

## Materials and methods

### Field sites

**Fig. 1:**
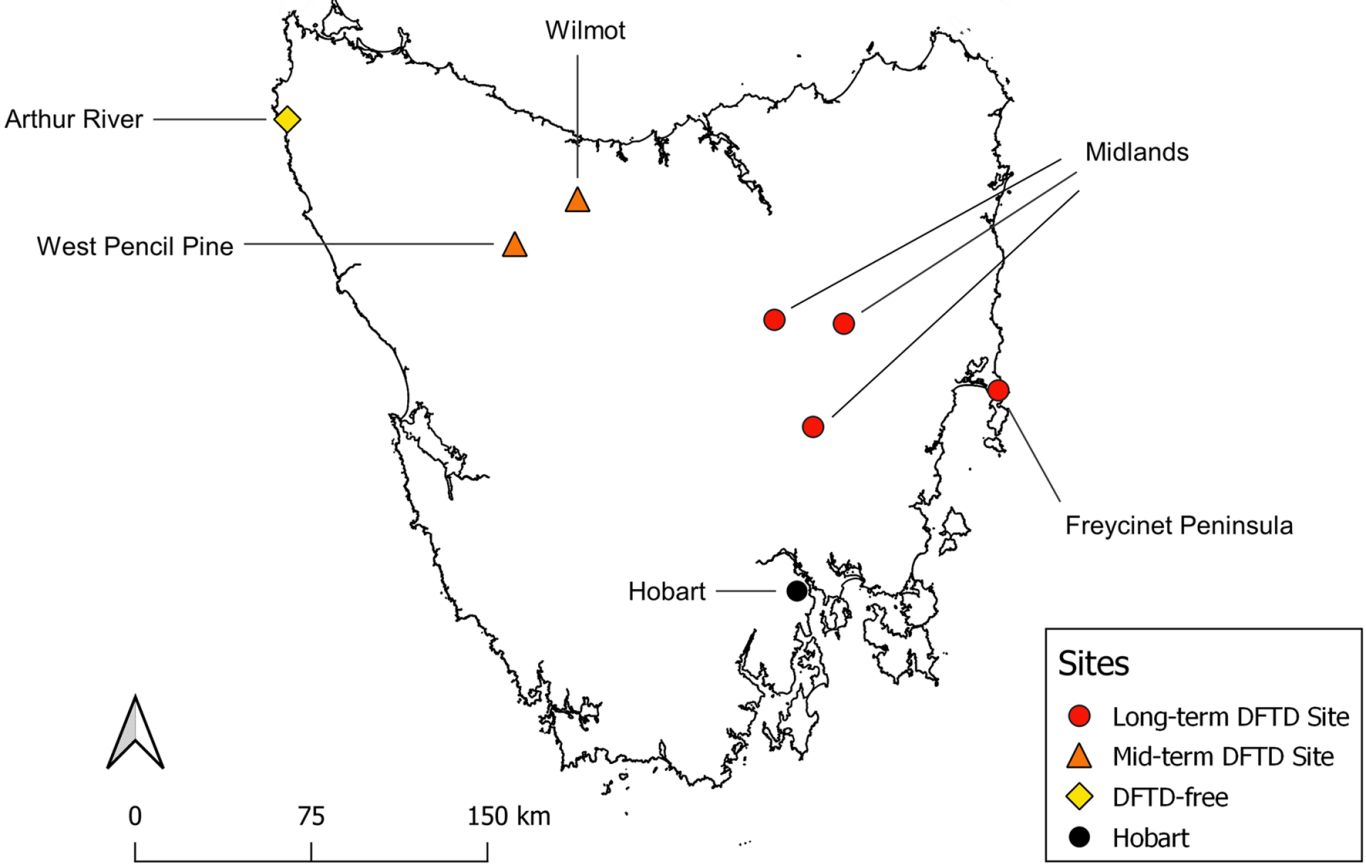
Locations of field sites in Tasmania at which Tasmanian devils and spotted-tailed quolls were sampled. Eastern quolls were sampled at West Pencil Pine. Hobart, the capital city of Tasmania, is shown for reference only (black circle). Study sites are categorised based on the estimated year DFTD arrived at the site, as a ‘Long-term DFTD Site’ (red circles), ‘Mid-term DFTD Site’ (orange triangles) or ‘DFTD-free’ (yellow diamond) (color figure online)

Our five field sites each had different DFTD infection histories and varied in their habitat composition (Fig. 1). They comprised: the Freycinet Peninsula (− 42.107E, 148.277S), the Midlands (the Forest, − 41.860E, 147.505S; Cressy, − 41.845E, 147.169S; Oatlands − 42.257E, 147.349S), West Pencil Pine (− 41.541E, 145.823S), Wilmot (− 41.377E, 146.152S), and Arthur River (− 41.055E, 144.679S). The Midlands site is a composite of three sites, which we have combined due to low sample sizes related to predator density, as well as geographic proximity and habitat similarity. DFTD was first recorded at Freycinet in 2001, in the Midlands between 2000 and 2003, at West Pencil Pine in 2006, and at Wilmot in 2008. Arthur River was free of DFTD at the time of sample collection. Freycinet is a coastal site predominantly of dry eucalypt woodland. The Midlands sites are farmland with fragmented patches of dry eucalypt woodland. Wilmot is predominantly a commercial eucalypt plantation within a farming landscape, while West Pencil Pine is a commercial eucalypt plantation situated close to a protected area. Arthur River is a coastal site composed predominantly of coastal scrub. Freycinet and Arthur River are largely protected national park or conservation areas.

### Data collection

#### Sample collection

During live trapping periods every three months from 2014 to 2019, we sampled a total of 515 individual animals and collected one whisker from each per sampling event. Our sample included Tasmanian devils (*n* = 384), spotted-tailed quolls (*n* = 109) and eastern quolls (*n* = 22; Table 1).

**Table 1:** Summary of the numbers of individual Tasmanian devils, spotted-tailed quolls and eastern quolls sampled at each site. One whisker was sampled per individual. Basal whisker samples from all individuals were used to calculate population-level isotopic niche breadths and overlap. A subset of these individuals’ whiskers were fully subsampled and used in our analysis of individual niche breadths. Sites where sample sizes are markedly different from those at other sites (e.g. the number of Tasmanian devils sampled in the Midlands) reflect low population density in these areas

Site	Species	Sample size		Sampling period
		Total	Individual study	
Freycinet peninsula	Tasmanian devil	106	14	2015–2018
	Spotted-tailed quoll	19	14	
Midlands	Tasmanian devil	17	17	2015–2017
	Spotted-tailed quoll	22	12	
West pencil pine	Tasmanian devil	88	14	2014–2019
	Spotted-tailed quoll	15	13	
	Eastern quoll	22	NA	
Wilmot	Tasmanian devil	94	14	2014–2017
	Spotted-tailed quoll	28	7	
Arthur river	Tasmanian devil	79	14	2015–2018
	Spotted-tailed quoll	25	11	
Total	Tasmanian devil	**384**	**73**	
	Spotted-tailed quoll	**109**	**57**	
	Eastern quoll	**22**	**NA**	
	All species	**515**	**130**	

Individual animals were identified using unique microchip transponders, and individual data including age, sex and weight were recorded at each capture event. Individuals were aged using tooth wear, which is accurate up to three years of age in Tasmanian devils (M.E. Jones, unpublished data). Whiskers were collected by cutting close to the skin with scissors and were stored in plastic bags at − 20 °C prior to laboratory preparation.

To enable the standardisation of predator isotopic data across sites, samples of muscle tissue from prey species were collected opportunistically. Muscle from the hind leg of road-killed wildlife was sampled from each study site and the immediate surrounding areas at least once per season throughout 2018. In the Midlands, muscle samples for brushtail possums *Trichosurus vulpecula*, Bennett’s wallabies, Tasmanian pademelons *Thylogale billardierii*, rabbits *Oryctolagus cuniculus* and hares *Lepus europaeus* were also obtained from landowners managing wildlife numbers under Crop Protection Permits. Muscle was taken as a representative sample of the bulk material likely to contribute to devil mass, although other tissues are also ingested. Muscle samples were stored at − 20 °C, then oven dried at 60 °C for 48 h and stored in sealed Eppendorf tubes.

#### Prey diversity surveys

To assess variation in composition of the prey assemblage, which may influence isotopic niche breadths of carnivores, we undertook a remote camera survey of the animal communities at each site. Cameras were deployed for 21 days in November–December 2018. Twelve cameras were installed at each site, except for the Freycinet Peninsula, which had 13 cameras, resulting in 273 camera-nights at Freycinet, and 252 at all other sites. Proportional coverage of different vegetation types was estimated for each site (TASVEG 3.0 GIS layer), and cameras were set in areas of each vegetation type in proportion to the extent of their coverage at the site (e.g. if a site was composed of 50% ‘agricultural, urban & exotic vegetation’, 6 out of 12 cameras were deployed in this habitat type). Camera locations could not be fully randomised due to constraints of access, so locations were randomly generated from a set of established Tasmanian devil trapping locations. Cameras were then placed at least 200 m away from the devil trap location, to reduce the possibility of predator or trapper presence/odour affecting remote camera visits. Remote cameras were deployed to primarily target small to medium mammals. As such, cameras were placed facing the ground with a vertical field of view; this orientation can improve detection frequencies of medium-sized marsupials and has been previously used to survey Australian mammals (Smith and Coulson 2012; McDonald et al. 2015). Cameras were tied to trees, approximately 1.5 m from the ground. Plastic bait canisters were filled with a bait mixture of honey, rolled oats and peanut butter, and tied to the same tree as the remote camera, in view of the camera, approximately 0.6 m off the ground.

### Sample preparation

In the laboratory, whiskers were rinsed in distilled water to remove surface contaminants and left to air-dry, before being placed in a freeze-dryer for 24 h. Whisker sections were chopped and placed into tin cups to a mass of 0.7 ± 0.1 mg for isotope analysis. To estimate population isotopic niche breadths and overlap, all whiskers were sampled by taking one section from the base, as this is the section grown closest to the time of collection. To quantify variation in individual isotopic niche breadths across sites, we took a time series of samples by sub-sampling along the entire whisker length for a subset of devils (*n* = 73) and spotted-tailed quolls (*n* = 57) (Table 1). Fully subsampling devil whiskers resulted in a median of 10 samples per whisker (range 3–30), while quoll whiskers resulted in a median of 4 samples per whisker (range 3–7). Oven-dried muscle samples of prey species were placed in a freeze-dryer for 24 h to remove any moisture built-up during storage and then homogenised using a pestle and mortar. Homogenised tissue was then placed into tin cups for isotope analysis, to a mass of 0.7 ± 0.1 mg.

### Stable isotope analysis

Stable isotope analyses were conducted using a Sercon INTEGRA2 elemental analyser-isotope ratio mass spectrometer at the University of Exeter, an Elementar Pyrocube Elemental Analyser linked to a Thermo-Fisher-Scientific Delta XP Plus isotope ratio mass spectrometer at the Stable Isotope Ecology Laboratory at the Scottish Universities Environmental Research Centre, and a Thermoquest EA1110 elemental analyser linked to a Europa Scientific 2020 isotope ratio mass spectrometer at Elemtex Ltd, Cornwall UK. Stable isotope ratios are expressed as delta (δ) values expressed in parts per thousand, or ‘per mil’ (‰) relative to international standards, according to:

$$\delta X = \left[ {\left( {\frac{{R_{sample} }}{{R_{standard} }}} \right) - 1} \right] \times 1000$$

where *X* = ^13^C or ^15^N, R_sample_ = heavy to light isotope ratio of the sample and R_standard_ = heavy to light isotope ratio of a standard (Vienna Pee Dee Belemnite for δ^13^C and atmospheric nitrogen for δ^15^N). In laboratory settings, materials calibrated against international standards are routinely used. Our samples were scale-corrected using the standards USGS40 and USGS41, and internal standards of the bovine liver, alanine, alagel, gel and glygel. Across laboratories and standards, average precision was 0.08‰ ± 0.01 (1 standard deviation ± standard error) for δ^13^C and 0.12‰ ± 0.01 for δ^15^N.

### Statistical analysis

Statistical analyses were conducted in R Version 3.5.2 (R Core Team 2018).

#### Stable isotope data standardisation

To facilitate the comparison of carnivore isotopic data across sites, we standardised the data according to variation in two herbivore species that form the bulk of the Tasmanian devil diet and reflect two different feeding styles (grazing and browsing): Bennett’s wallabies and Tasmanian pademelon (Jones and Barmuta 1998). Muscle samples of both species were collected at all sites (*n* per species at each site > 6, total *n* = 116). We then combined their isotopic values at each site and calculated mean δ^13^C and δ^15^N per site. Taking one site, Wilmot, as our baseline, we then adjusted predator and prey δ^13^C and δ^15^N values at other sites by the difference between the mean δ^13^C and δ^15^N of wallabies and pademelons at the relevant site and the mean δ^13^C and δ^15^N of wallabies and pademelons at Wilmot. This does not fully characterise the isotopic baseline across all sites as the prey assemblage is likely to be more isotopically diverse in some sites than others, however, this method at least allows us to standardise values of δ^13^C and δ^15^N relative to a common baseline.

#### Population niche breadth and overlap

We estimated the isotopic niche breadths of Tasmanian devil, spotted-tailed quoll and eastern quoll populations using two methods: Standard Ellipse Areas corrected for sample breadth (SEA_C_), and Standard Ellipse Areas calculated using Bayesian inference (SEA_B_) (Jackson et al. 2011). These two metrics return similar information, though SEA_B_ provides uncertainty estimates and is more robust to small sample sizes. While we use SEA_C_ for visualisation, we rely on SEA_B_ for further quantitative analysis.

Quoll whiskers are finer in structure than devil whiskers, therefore to achieve the target weight for an isotopic sample (0.7 ± 0.1 mg), a longer section of quoll whisker was generally sampled in comparison to devils. This sampling strategy could result in devil and quoll isotopic niches reflecting diet integrated over different periods of time, particularly if devil and quoll whiskers have different growth rates. To test whether our SEA_B_ estimates are sensitive to the amount of whisker sampled, we recalculated SEA_B_ for each site, using the subset of devils and spotted-tailed quolls fully subsampled for individual niche analysis. SEA_B_ was first calculated using the basal sections of individuals’ whiskers alone, and then again using an average of the two most basal whisker sections per individual. There was little difference between SEA_B_ estimates calculated using one and two samples per individual for both species, so we proceeded with our population niche estimates and comparisons using basal samples.

The overlap between isotopic niches of species dyads at each site was calculated using Bayesian methods in SIBER (Jackson et al. 2011), by estimating the overlap between each dyad of ellipses over 500 draws from posterior estimates, which was then expressed as the proportion of the non-overlapping areas of the ellipses.

#### Drivers of population niche breadth and overlap

To investigate the drivers of isotopic niche breadth of Tasmanian devil and spotted-tailed quoll populations, and niche overlap between the two species, we built two sets of models, using the modal SEA_B_ estimates of Tasmanian devil and spotted-tailed quolls (*n* = 10), and the modal overlap between the two species at each site (*n* = 5) as response variables. We fitted linear models, separately analysing the effect of species, as there may be consistent differences in niche breadth between devils and spotted-tailed quolls; the time since DFTD was first recorded at each site (categorised as long-term [2003 and before], mid-term [post 2003] and DFTD-free); road cover per km^2^, as road cover may increase carrion availability; the percentage of the site area composed of habitat modified by people, as community niche dynamics can be influenced by human disturbance; and Shannon’s diversity of functional prey groups, to investigate whether variation in prey diversity and evenness drove variation in niche breadth. We also fitted a linear model regressing the SEA_B_ of Bennett’s wallabies and Tasmanian pademelons (combined) against the predator SEA_B_ metrics, to assess whether devil and quoll niche breadths are driven by the variation in isotopic baseline within sites, rather than dietary variation. We applied a Bonferroni correction to correct for multiple hypothesis testing and reduce the chance of Type 1 errors.

The percentage of human-modified habitat (including farmland, commercial eucalypt plantations and private residential properties) and road cover (per km^2^) at each site were calculated in QGIS using open-source LIST Transport Segment and TASVEG 3.0 GIS layers. Home ranges of predators caught towards the edges of our trapping areas may extend beyond the trapping boundaries. To account for this, we added a buffer of 3.22 km (unless extent was restricted by coastline) around our trapping area for each site, based on the radius of the mean 95% kernel density estimate recorded for female devils on the Freycinet Peninsula prior to the first recorded DFTD infection in the area (Comte et al. 2020). We used female home ranges as little data was available on male home range size (Comte et al. 2020). Transport segments were filtered to remove non-road segments and restricted tracks. We then extracted road length and habitat area data from within the trapping area and surrounding buffer.

To characterise differences in the prey assemblages across sites, we calculated Shannon’s Diversity Index using remote camera survey data. Animal detections of the same species were treated as unique and included in the analysis if they occurred over 20 min apart. We grouped species into functional groups, as differences in functional diversity rather than species diversity may have the strongest effect on the isotopic and ecological diversity of predator niches. These groups included: large exotics (fallow deer *Dama dama*, sheep *Ovis aries*), large marsupial herbivores (eastern grey kangaroo *Macropus giganteus*, common wombat), medium macropods (Bennett’s wallaby, Tasmanian pademelon), medium arboreal marsupials (brushtail possums, ringtail possums *Pseudocheirus peregrinus*), medium terrestrial marsupials (long-nosed potoroo *Potorous tridactylus*, eastern bettong *Bettongia gaimardi*, southern brown bandicoot *Isoodon obesulus*), monotremes (short-beaked echidnas *Tachyglossus aculeatus*), small mammals (eastern pygmy possum *Cercartetus nanus*, sugar glider *Petaurus breviceps*), native rodents (long-tailed mouse *Pseudomys higginsi*, Australian swamp rat *Rattus lutreolus*, broad-toothed rat *Mastacomys fuscus*), non-native rodents (black rat *Rattus rattus*, house mouse *Mus musculus*), birds and reptiles. Birds and reptiles were not resolved further due to low sample sizes, and, as our remote camera were set up to target mammals, there will likely be biases in a sampling of birds and reptiles captured in our survey, due to differences in habitat and behaviour.

#### Individual isotopic niche breadths

To quantify individual isotopic niche breadths of devils and quolls across sites, we calculated individual standard ellipse areas for 130 individuals for which whiskers were fully subsampled (Table 1). To quantify variation in the extent of individual specialisation between species and sites, we then calculated a relative niche area index for each individual by expressing their individual isotopic niche breadth (standard ellipse area) as a proportion of the total isotopic niche area of its species at the relevant site, henceforth ‘combined niche’ breadth, using the siberKapow function of the R package *SIBER* (Jackson and Parnell 2017). Here, the total combined niche breadth per site was measured as the total area occupied by the individual isotopic niches of all individuals of that species also fully subsampled for individual niche analysis. This measure was positively correlated with the SEA_B_ mode estimates used to quantify population niche breadths and ecological drivers of niche variation (Spearman’s rho = 0.79, *p* = 0.01). If relative individual isotopic niche breadth decreases with increasing combined niche area, this would suggest population niche expansion via increased individual specialisation, as individuals occupy a small proportion of the total available isotopic niche space. Conversely, if absolute individual isotopic niches increase with combined niche but the relative individual niche metric remains constant, or if the relative individual niche metric increases due to an increased absolute niche with reduced or constant combined niche breadth, this would suggest population niche expansion via increased generalism in individuals.

To analyse whether relative individual isotopic niche breadth varied between sites and with combined isotopic niche breadth, we regressed relative individual isotopic niche breadths against the fixed variables: species, number of whisker sections used to generate that individual’s niche breadth (as low sample size may result in underestimates of isotopic niche breadth), combined isotopic niche area, and site. As our response variable data were continuous, proportional data between 0 and 1, we chose a beta regression model framework (Douma and Weedon 2019) with a logit link function, using the R package *betareg* (Cribari-Neto and Zeileis 2010).

To analyse whether individual absolute isotopic niche breadth varied between sites and with combined isotopic niche breadth, we regressed individual isotopic niche breadths against the same fixed variables as above: species, the number of whisker samples obtained, the combined isotopic niche area, and site. As our response variable data were zero-bounded and positively skewed, we used a Gamma error structure with an identity link function.

## Results

### Population niche breadth and overlap

**Fig. 2:**
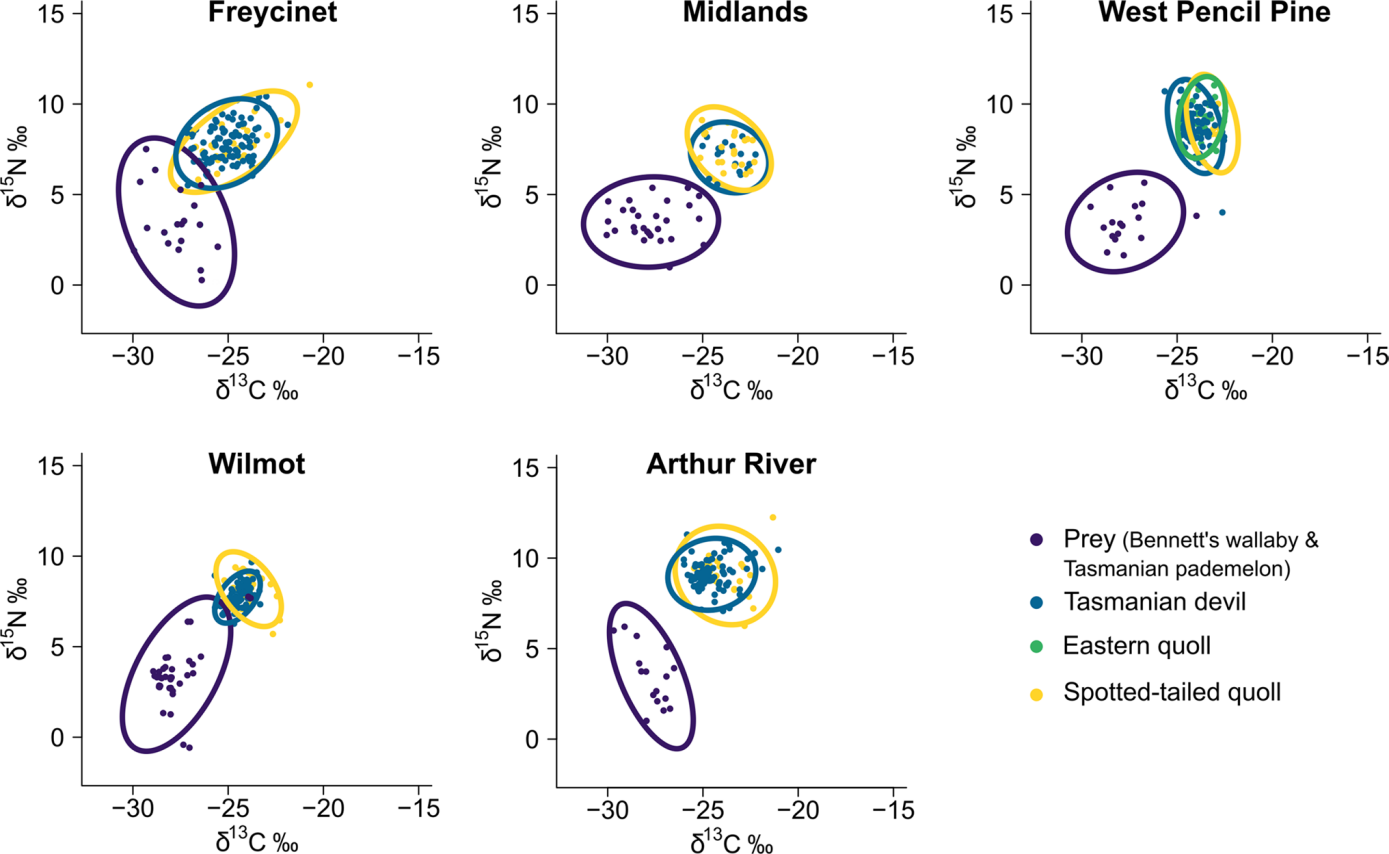
Isotopic niches, represented as standard ellipse areas estimated using δ^13^C and δ^15^N values, for Tasmanian devils (blue), spotted-tailed quolls (yellow), eastern quolls (green) and their prey species (purple) across five study sites in Tasmania. Sites are presented in order of time since DFTD is estimated to have arrived in the resident population, from Freycinet (top left) to Arthur River (bottom middle). Prey standard ellipse areas are provided to allow comparison of carnivore ellipse areas with general isotopic baseline variability (color figure online)

Combining all standardised data, average δ^13^C values were similar for eastern quolls and spotted-tailed quolls (eastern quoll δ^13^C mean = − 23.72; spotted-tailed quoll δ^13^C mean = − 23.90), but were lower for Tasmanian devils (δ^13^C mean = − 24.80), while average δ^15^N values were highest for eastern quolls (δ^15^N mean = 9.26), but similar for spotted-tailed quolls and Tasmanian devils (spotted-tailed quoll δ^15^N mean = 8.20; Tasmanian devil δ^15^N mean = 8.16) (Table 2). Across the five study sites, Tasmanian devils and spotted-tailed quolls had similar isotopic niche breadths (Tasmanian devil SEA_B_ mode range 0.78–3.28; spotted-tailed quoll SEA_B_ mode range 1.54–3.87) (Fig. 2). Neither species had a consistently larger niche than the other; Tasmanian devils had a larger isotopic niche breadth (SEA_B_ mode) at the Midlands and West Pencil Pine, while spotted-tailed quolls had larger isotopic niche breadths at Freycinet, Wilmot and Arthur River. At West Pencil Pine, eastern quolls had a smaller isotopic niche breadth (SEA_B_ mode = 1.41, 95% CI = 0.88–2.15) than devils or spotted-tailed quolls (devil SEA_B_ mode = 1.86, 95% CI = 1.50–2.28; spotted-tailed quoll SEA_B_ mode = 1.75, 95% CI = 1.02–3.05). The extent of isotopic niche overlap between devils and spotted-tailed quolls was lowest at Wilmot (Bayesian modal overlap estimate = 0.30, 95% CI = 0.20–0.41), and largest at Freycinet (Bayesian modal overlap estimate = 0.67, 95% CI = 0.51–0.81). At West Pencil Pine, the isotopic niche of eastern quolls overlapped slightly more with devils (overlap mode = 0.49, 95% CI = 0.32–0.68) than spotted-tailed quolls (overlap mode = 0.45, 95% CI = 0.23–0.66), and devils and spotted-tailed quolls overlapped the least (overlap mode = 0.37, 95% CI = 0.18–0.60).

**Table 2:** A summary of mean δ^13^C and δ^15^N of Tasmanian devils, spotted-tailed quolls, eastern quolls and prey species (Bennett’s wallabies and Tasmanian pademelons) at our five study sites, before and after standardisation. Data were standardised by adjusting prey and consumer data by the difference between the mean δ^13^C and δ^15^N of prey at the relevant site and those observed at Wilmot, which was chosen as an isotopic baseline (data in bold)

Site	Species	Unstandardised data		Standardised data	
		Mean δ^13^C	Mean δ^15^N	Mean δ^13^C	Mean δ^15^N
Freycinet peninsula	Tasmanian devil	− 22.67	8.86	− 25.46	7.64
	Spotted− tailed quoll	− 21.69	9.07	− 24.47	7.85
	Prey	− 24.92	4.70	− 27.71	3.48
Midlands	Tasmanian devil	− 22.82	9.03	− 23.94	7.15
	Spotted− tailed quoll	− 22.48	9.16	− 23.59	7.28
	Prey	− 26.59	5.36	− 27.71	3.48
West pencil pine	Tasmanian devil	− 23.97	6.89	− 24.34	8.93
	Spotted− tailed quoll	− 22.83	7.12	− 23.21	9.15
	Eastern quoll	− 23.35	7.23	− 23.72	9.26
	Prey	− 27.34	1.45	− 27.71	3.48
Wilmot	Tasmanian devil	− 24.75	7.74	− 24.75	7.75
	Spotted− tailed quoll	− 24.28	8.04	− 24.28	8.04
	**Prey**	**− 27.71**	**3.48**	**− 27.71**	**3.48**
Arthur river	Tasmanian devil	− 24.37	8.40	− 25.01	9.07
	Spotted− tailed quoll	− 23.20	8.12	− 23.85	8.79
	Prey	− 27.06	2.81	− 27.71	3.48
Combined	Tasmanian devil	− 23.72	8.16	− 24.80	8.16
	Spotted− tailed quoll	− 22.78	8.35	− 23.90	8.20
	Eastern quoll	− 23.35	7.23	− 23.72	9.26
	Prey	− 27.71	3.48	− 27.71	3.48

### Drivers of population niche breadth and overlap

**Fig. 3:**
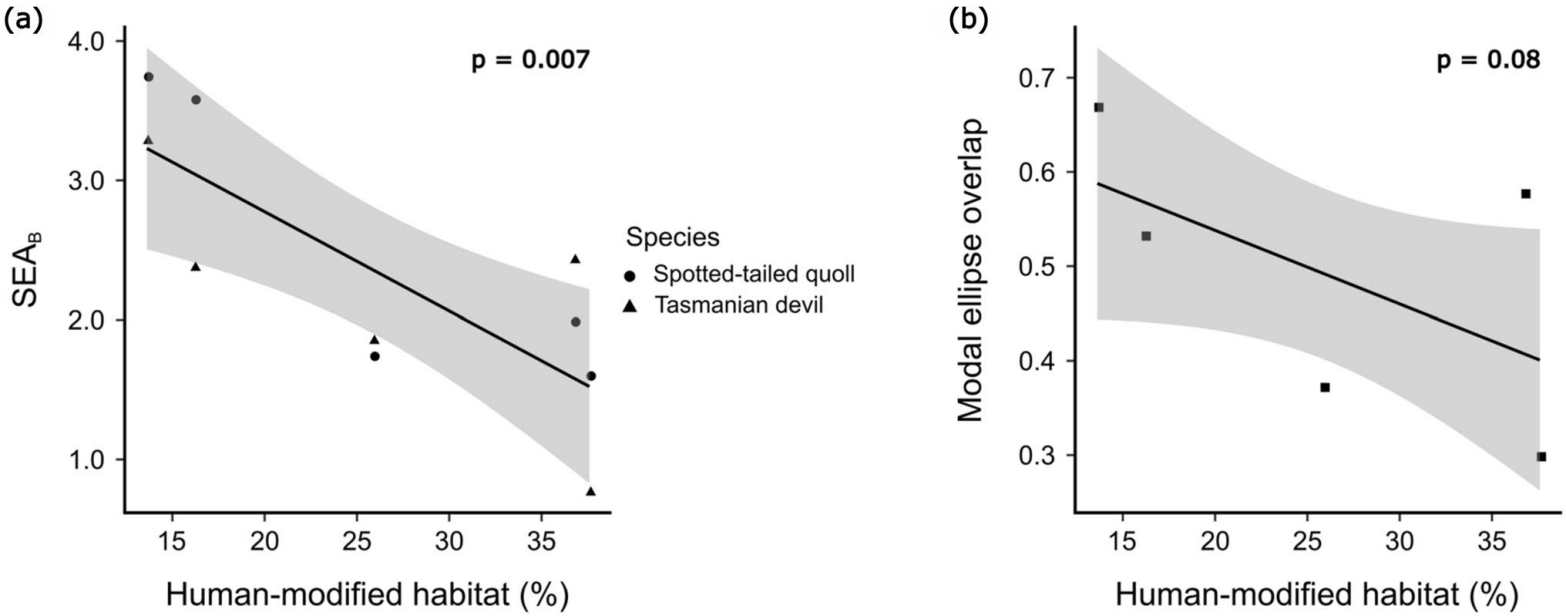
Relationships between the amount of human-modified habitat at each of five sites (%) with the isotopic niche breadths of Tasmanian devils and spotted-tailed quoll populations, and the amount of niche overlap between the two species, at each site. Isotopic niche breadths of are represented by modal Bayesian standard ellipse area (SEA_B_ in panel (**a**)), and niche overlap between devil and quoll isotopic niches at each site was expressed as the mode of the Bayesian-estimated extent of niche overlap (as a proportion of the total non-overlapping area) (panel (**b**))

The percentage of human-modified habitat at each site had a significant effect on the variation in population isotopic niche breadths in Tasmanian devils and spotted-tailed quolls (*p* = 0.007, alpha = 0.008, Table 3) but not the extent of overlap between them (*p* = 0.08, alpha = 0.008, Table 3). At sites with a higher percentage of human-modified habitat, including the Midlands and Wilmot (Table 3), both devils and spotted-tailed quolls had smaller isotopic niches than more natural sites such as Freycinet and Arthur River (Fig. 3). Contrary to our predictions, time since DFTD arrival, used as a proxy for level of devil decline, had no effect on the isotopic niche breadths or niche overlap of devils or spotted-tailed quolls (Table 3).

**Table 3:** Summary of the ecological variables used to predict the local sampled population isotopic niche breadths of Tasmanian devils and spotted-tailed quolls at five sites (SEA_B_ mode) and isotopic niche overlap of the two species at each site (modal overlap estimate), and the p-values related to each test (p-values in bold). Ecological predictors included species, the amount of time since DFTD arrived at the site (DFTD category), the percentage of human-modified habitat at each site, the amount of road cover, the functional prey diversity (Shannon’s diversity index) and the isotopic niche breadth of prey species (Prey SEA_B_ mode) at each site. As each predictor was run as a separate model for each of our two response variables (6 models per response variable), our critical p-value was 0.008

	Species	DFTD category	Human-modified habitat (%)	Road cover (km^2^)	Shannon’s diversity index	Prey SEA_B_ mode
Freycinet	NA	Long-term DFTD region	13.62	1.48	0.93	6.68
Midlands	NA	Long-term DFTD region	36.75	0.83	1.20	4.51
West pencil pine	NA	Mid-term DFTD region	25.87	1.18	2.13	4.19
Wilmot	NA	Mid-term DFTD region	37.59	1.96	1.36	5.07
Arthur river	NA	DFTD-free	16.18	1.60	1.55	3.58
SEA_B_ mode *p* value	**0.55**	**0.74**	**0.007**	**0.71**	**0.22**	**0.52**
Ellipse overlap *p* value	**1.00**	**0.20**	**0.08**	**0.23**	**0.05**	**0.36**

The SEA_B_ mode of prey species (Bennett’s wallaby and Tasmanian pademelon) at each site also had no effect, suggesting that differences in the isotopic variability of available prey resources does not explain variation in predator niche breadths nor overlap. The effects we demonstrate are therefore likely ecological, rather than an artefact of different isotopic baselines of different sites.

### Individual isotopic niche breadths

**Fig. 4:**
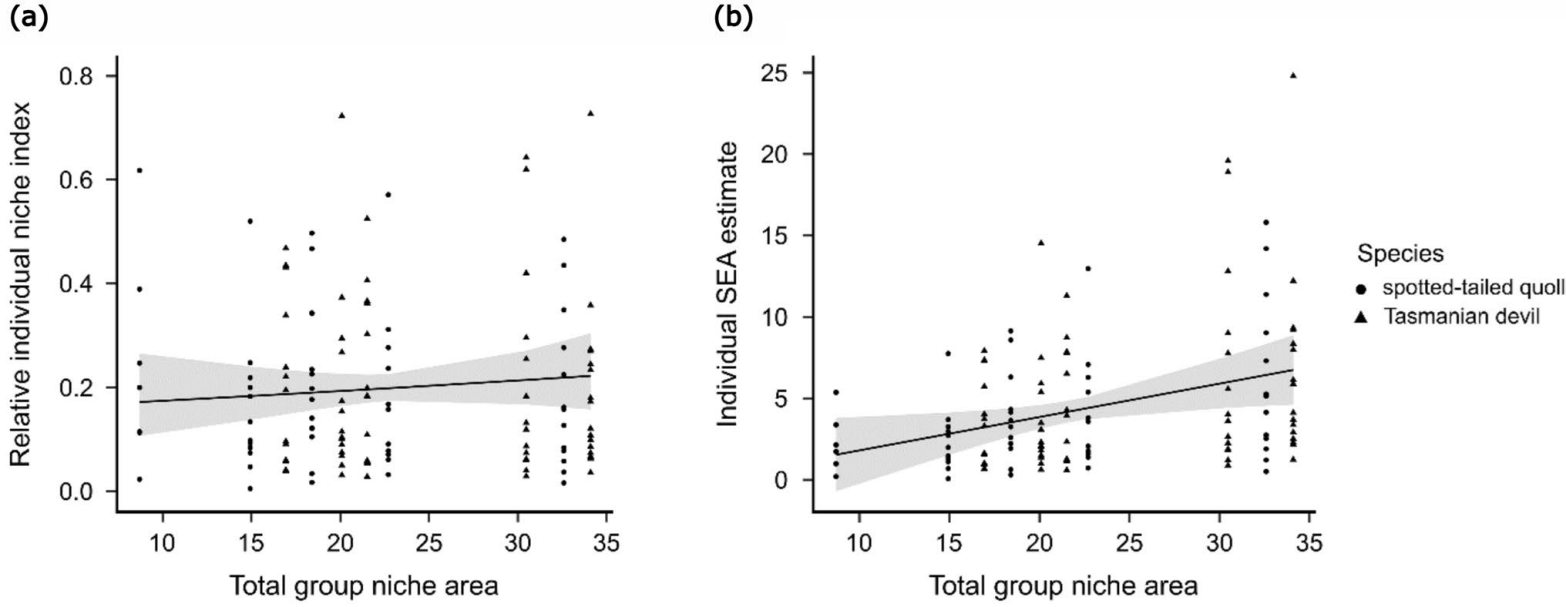
The model-fitted relationship between total combined isotopic niche area (the total area of all individual ellipses for each species/site group) and individual devil and quoll relative individual niche index estimates (panel (**a**)) and individual standard ellipse area estimates (panel (**b**)). Observed relative individual niche index estimates and individual standard ellipse area estimates are plotted. Relative individual niche index estimates are individual SEA estimates expressed as a proportion of the combined niche area for the relevant species/site group

The total combined isotopic niche breadth (of individuals of a single species at a single site) was a significant positive predictor of absolute individual niche breadth (estimate = 0.21, *p* = 0.01, Fig. 4). Relative individual niche breadth did not vary significantly between devils and spotted-tailed quolls or by site, and did not vary by total combined isotopic niche breadth (Table 4). No other terms in this model contributed significantly to explaining variance.

**Table 4:** Summary of the results from models investigating the drivers of relative individual niche breadths and individual standard ellipse areas of Tasmanian devils and spotted-tailed quolls. Relative individual niche breadth was fitted in a beta regression framework and individual standard ellipse area as a generalised linear model with a Gamma error family and identity link function. No fitted predictors had a significant relationship with relative individual niche breadth (individual ellipse areas expressed as a proportion of the total combined niche area). Total combined niche area had a significant positive relation with individual ellipse area (p-value in bold)

Response variable	Model variables	Estimate	Standard error	Lower confidence interval	Upper confidence interval	z/t value	p value
Relative individual niche breadth	Intercept	− 1.81	0.42	− 2.63	− 0.99	− 4.34	< 0.001
	Species (devil)	− 0.25	0.24	− 0.72	0.21	− 1.06	0.29
	Number of whisker sections	0.03	0.02	− 0.006	0.07	1.61	0.11
	Combined niche area	0.01	0.02	− 0.02	0.05	0.68	0.50
	Site (Freycinet)	− 0.13	0.31	− 0.73	0.48	− 0.41	0.68
	Site (Midlands)	− 0.06	0.25	− 0.55	0.43	− 0.24	0.81
	Site (West Pencil Pine)	0.12	0.23	− 0.34	0.58	0.51	0.61
	Site (Wilmot)	0.20	0.29	− 0.36	0.76	0.72	0.48
Individual ellipse area	Intercept	− 0.66	1.76	− 4.22	2.87	− 0.37	0.71
	Species (devil)	− 0.47	1.09	− 2.61	1.72	− 0.43	0.67
	Number of whisker sections	0.06	0.10	− 0.11	0.29	0.67	0.51
	**Combined niche area**	**0.21**	**0.08**	**0.05**	**0.38**	**2.47**	**0.01**
	Site (Freycinet)	0.01	1.52	− 3.18	3.36	0.004	1.00
	Site (Midlands)	− 0.23	0.92	− 2.21	1.61	− 0.25	0.80
	Site (West Pencil Pine)	0.42	0.96	− 1.59	2.42	0.44	0.66
	Site (Wilmot)	0.55	1.08	− 1.71	2.70	0.51	0.61

## Discussion

Isotopic niche breadths of Tasmanian devils and spotted-tailed quolls estimated using δ^13^C and δ^15^N values of whiskers are smaller in human-modified landscapes (Fig. 3). This variation in local population isotopic niche breadth in different landscapes appears to be driven by variation in individuals’ isotopic niche breadths. However, individuals’ niches remain consistent as a proportion of their total combined niche, suggesting limited variation in individual specialisation between populations.

Our study of carnivore isotopic niches across diverse habitat types and ecological contexts adds further weight of evidence that top-down community effects may vary with ecosystem context (Finke and Denno 2004; Elmhagen and Rushton 2007; Kuijper et al. 2016; Wang et al. 2020). We found that the time since DFTD arrived at a site did not influence devil or spotted-tailed quoll isotopic niches, or the degree of isotopic niche overlap between the two species. This is despite multiple previous studies demonstrating devil decline has precipitated trophic cascades and changes in spotted-tailed quoll behaviour, including an increase in scavenging in areas where devils have declined (Hollings et al. 2014; Andersen et al. 2017; Cunningham et al. 2018). Tasmanian devils may exhibit top-down controls in natural landscapes, while bottom-up factors play a greater role in human-modified landscapes. For example, a strong positive effect of devil decline on carcass persistence and mesopredator scavenging behaviour has been revealed across environmentally comparable sites of two natural habitat types, with minimal human disturbance (Cunningham et al. 2018); clearly, across these landscapes, devils exhibit top-down effects on sympatric competitors. However, while devil decline has generally caused cascades in Tasmania via increased feral cat occurrence, this effect was dampened in areas with low rainfall and, critically, in areas with relatively high human disturbance, where bottom-up factors such as prey availability had the greatest effect on cats (Hollings et al. 2014). A more detailed understanding of the relationship between top-down and bottom-up impacts on carnivore isotopic niches in this system would require a broader range of study sites with replication of multiple habitat types, however, our results suggest that across several habitat types, anthropogenic modification of landscapes affects the realised niches of devils and spotted-tailed quolls to a greater extent than the ecological and competitive effects of top carnivore decline.

Our results contribute to the emerging evidence that human-modified habitats influence the resource use, diet and isotopic niches of carnivores, and demonstrate that this effect is not universal in its direction. Most related studies show that carnivores in human-modified landscapes increase their use of human resource subsidies, including livestock and other domestic species, carcasses and food waste (Newsome et al. 2015a, b), resulting in broader population isotopic niches (Magioli et al. 2019), and/or increased dietary overlap between carnivore species (Newsome et al. 2014; Manlick and Pauli 2020). In contrast, we report smaller isotopic niches of marsupial carnivores in human-modified landscapes, with no change in the degree of isotopic niche partitioning. We suggest that differences in resource availability represented by human-modified landscapes in different ecosystems and taxonomic differences may result in different effects on carnivore isotopic niches. For example, in a systematic review of the ecological impacts of human resource subsidies on carnivores, Newsome et al. (2015a, b) found variations in patterns of human-related resource consumption between canids, felids and ursids. It is possible that dasyurids, such as devils and quolls, simply do not exploit, or gain access to, human resource subsidies to the same degree as other taxonomic groups. Additionally, it is likely that in Tasmanian landscapes, human resource subsidies do not offset native prey losses associated with landscape modification. As a result, carnivorous marsupials would be competing in a low-quality habitat, relative to native landscapes, driving niche contraction. The agricultural Midlands region of Tasmania is highly deforested and fragmented (Michaels et al., 2010), which has led to widespread declines of native species and the domination of a relatively small number of invasive species, such as feral cats, black rats, and noisy miners *Manorina melanocephala* (Gardiner 2018; Bain et al. 2020; Hamer et al. 2021; Jones et al. 2021). Our other most modified landscape, Wilmot, is largely composed of a eucalypt planation monoculture. The breadth of prey resources available to native carnivores in these areas is expected to be low compared to more natural landscapes.

The niche variation hypothesis predicts that release from interspecific competition leads to population niche expansion, potentially via increased between-individual variation (Van Valen 1965). This has been supported by multiple empirical studies (Araujo et al. 2009; Darimont et al. 2009; Bolnick et al. 2010). However, while we found that the individual niche breadths of Tasmanian devils and spotted-tailed quolls generally contract in human-modified landscapes, driving population isotopic niche contraction, the degree of individuals’ specialisation relative to one another remains constant. Several mechanisms can drive reduced isotopic niche breadths of individuals and populations. First, isotopic niche breadths of prey species may be narrower in some landscapes. We tested for this and found no effect of wallaby and pademelon isotopic niche areas upon carnivore population isotopic niches. Second, predator isotopic niches could be genuinely constrained or relaxed by variation in ecological opportunity across landscapes with varying degrees of human modification. Even with this variation, competitive conditions within conspecific populations may remain constant enough that the proportional overlap of conspecific individuals does not change. If prey resource diversity reduces in human-modified landscapes but resources are still abundant, this could constrain population niche breadths but would allow individuals to maintain a degree of differentiation from conspecifics. This could occur either through variation in the proportions of different prey items ingested or through variation in foraging locations resulting in isotopic variation at a scale we are not able to assess. In agricultural areas of Tasmania, herbivores are regularly culled, with carcasses left in open ground, providing a narrow but plentiful resource base.

Although isotopic niches are related to ecological niches, they are not equivalent and careful interpretation is necessary (Bearhop et al. 2004; Jackson et al. 2012). Changes in the trophic niche of devils and quolls might occur following devil decline, without the effect being discernible using isotopic analyses, particularly when compared to the effect of human-modified habitat. Therefore, we highlight that our results do not suggest devil decline has no impact on the trophic behaviour or niche of the devil or spotted-tailed quoll populations. Furthermore, when constructing individual isotopic niches, care must be taken as the temporal scale at which a chosen tissue, in our case whiskers, integrates may not capture differences in the time scale of dietary variation between sites. If variation occurs over shorter time scales in some sites than others, isotopic variation in whiskers from these sites may be averaged out, appearing less variable. For example, eastern quoll whiskers are finer and shorter, than either the devil or spotted-tailed quoll whiskers, therefore a longer length of the whisker is needed to create one sample for isotope analysis. Therefore, any isotopic variation along the whisker could become averaged out in the process of returning single values for δ^13^C and δ^15^N, resulting in a small isotopic niche breadth even if a dietary variation is broad. We were able to test whether our devil and spotted-tailed quoll isotopic niche estimates were sensitive to sample length by varying the length of the whisker averaged to create our estimates, but this was not possible for eastern quolls as their whiskers generally only yielded a single sample. Therefore, we would caution against confirming that Eastern quolls have a consistently narrower isotopic niche than spotted-tailed quolls or devils based on our study. Nevertheless, our results provide valuable insight into the trophic dynamics of carnivorous marsupials across a range of habitat and species decline contexts.

The influence top carnivores can have on community structure increases the importance of understanding not just the ecological consequences of carnivore decline, but how these interact with other global phenomena such as anthropogenic habitat change and fragmentation. Here, we have shown that isotopic niche breadths of Tasmanian devils and spotted-tailed quolls are sensitive to human disturbance across a variety of habitat types, but do not vary according to disease history, suggesting bottom-up forces have a stronger effect on niche partitioning in disturbed areas than the presence or absence of top-down control by Tasmanian devils. This contributes to our knowledge of the complex interactions between top-down and bottom-up forcing in carnivore guilds. Furthermore, we demonstrate that the directionality of ecological effects of processes such as land-use change may vary according to the specific ecologies and opportunities of species, populations and individuals, and should not be assumed to be consistent.

## Data Availability

Data is available at the Dryad data repository 10.5061/dryad.qnk98sfqs.

## Code availability

Code is available at the Dryad data repository 10.5061/dryad.qnk98sfqs.
